# Assessing basic/fundamental psychological need fulfillment: systematic mapping and review of existing scales to foster cumulative science

**DOI:** 10.3389/fpsyg.2024.1427478

**Published:** 2024-09-30

**Authors:** Tjaša Kermavnar, Andreja Avsec, Siyuan Huang, Pieter M. A. Desmet

**Affiliations:** ^1^Department of Human-Centered Design, Faculty of Industrial Design Engineering, Delft University of Technology, Delft, Netherlands; ^2^Department of Psychology, Faculty of Arts, University of Ljubljana, Ljubljana, Slovenia

**Keywords:** psychological needs, need satisfaction, need frustration, psychometric scale development, psychometric scale validation

## Abstract

**Background:**

Because the fulfillment of basic/fundamental psychological needs affects people’s motivation and well-being, measuring the degree to which these needs are met is of interest to researchers across various domains. Although numerous self-assessment tools have been developed, no recent comprehensive reviews exist, hindering cumulative scientific progress. The present systematic review aimed to identify and analyze the main approaches to developing self-report scales for assessing basic/fundamental psychological need fulfillment. The objective is to inform readers interested in selecting instruments for their studies and those intending to develop new scales.

**Methods:**

Following PRISMA, we conducted a search of Scopus, Web of Science, PubMed, and ProQuest in August 2023. The following information was extracted from eligible studies: Scale name, abbreviation, theoretical basis, application domain, final scale construction, scale development and validation methodology, and citation count.

**Results:**

Our search identified 32 primary studies, in which 31 original scales were developed and validated, and 89 secondary studies that aimed to modify these original scales. The predominant theoretical basis was Self-Determination Theory, although eight scales were based on alternative need typologies. The scales were either domain-general or specific to contexts such as work, education, or exercise/sports contexts. While most were designed to measure need satisfaction, some also addressed need support, frustration, and thwarting.

**Conclusion:**

Despite significant efforts in developing, adapting, and applying scales to measure need fulfillment, we found several issues resulting from diverse perspectives on conceptualizing psychological needs and need typologies, discordant approaches in developing and validating measures, and other inconsistencies that should be acknowledged and addressed in future research.

## 1 Introduction

Needs play a crucial role in human functioning, underpinning almost all of our daily goals and actions. For decades, numerous distinguished psychologists such as Maslow (1943), Murray (1938), and more recently Deci and Ryan (2000), have advanced the idea that humans share a common set of basic or fundamental needs, the fulfillment of which is essential for personal growth and wellbeing.

Several theories have been proposed to form need typologies, with foundational work that can be traced back to Maslow’s (1943) Hierarchy of Needs and Murray’s (1938) theory of needs. One of the most prominent frameworks is the Basic Psychological Need Theory (BPNT; Vansteenkiste et al., 2020), which is part of the broader Self-Determination Theory (SDT; Ryan and Deci, 2017). BPNT identifies three basic psychological needs: Autonomy, Competence, and Relatedness. Alongside BPNT, other notable theories include the Existence, Relatedness, and Growth (ERG) Theory (Alderfer, 1969), the Manifest Needs Theory (MNT; McClelland, 1987), the interdisciplinary Detachment-Recovery, Autonomy, Mastery, Meaning, and Affiliation (DRAMMA) model (Newman et al., 2014), and the Psychology of Working Theory (PWT; Duffy et al., 2016).

Early renditions of need systems, such as those developed by Murray (1938) and Maslow (1943), classified human needs into two categories based on how essential they were for survival. The first includes physiological (physical/biological/bodily/viscerogenic) needs, which were considered to be “primary,” “fundamental,” or “basic,” as their fulfillment is essential for survival. The second includes psychological needs, which were deemed “secondary” and prioritized after existential threats are resolved, as they contribute to *thriving* rather than mere *survival* (Welzel, 2013). Although the concept of basic bodily needs is relatively easy to grasp, delineating a basic psychological need from a non-basic one remains challenging. Consequently, identifying when and how particular psychological needs are met or unmet poses an even greater challenge.

The diversity of need typologies illustrates a current lack of consensus of what constitutes a *fundamental* psychological need and how this differs from non-basic needs. Over the years, various criteria have been proposed to address this issue. One of the earliest efforts was published by Baumeister and Leary (1995), comprising nine principles. Later, Ryan and Deci (2017) introduced new criteria to distinguish between basic (“growth”) from non-basic (“deficit”) psychological needs. More recently, Vansteenkiste et al. (2020) presented a more refined list of requirements, consisting of five “basic” and four “associated” criteria.

Another debate revolves around the relationship between basic psychological needs. Maslow (1943) famously proposed a hierarchical relationship, where “higher-level” needs emerge only once “lower-level” needs are satisfied. ERG similarly posits that needs can be interrelated, while Baumeister and Leary (1995) and early SDT researchers held an opposing view, proposing that needs can operated independently. Recent research, however, has indicated possible relationships among the SDT needs. For instance, studies by Baard et al. (2004) and Adie et al. (2012) found that autonomy support positively influences the fulfillment of all three SDT needs.

There are substantial differences in how psychological needs have been operationalized in psychometric scales, which in turn influences how item pools are formulated (see Table 1 for examples). One key distinction is between scales based on a uni-dimensional versus a two-dimensional model of need fulfillment. Early psychometric scales followed a unidimensional model, where a need was either fulfilled or not, measured along a single continuum. These scales focused on the “bright” side of human functioning—the extent to which needs were satisfied (e.g., Reeve and Sickenius, 1994). However, about a decade ago, a conceptual shift occurred toward a two-dimensional perspective, recognizing need frustration as distinct from low need satisfaction, representing the “dark” side of human functioning (Vansteenkiste et al., 2020). The model presented by Vansteenkiste and Ryan (2013) explains the asymmetry between these experiences: While need frustration inherently indicates the absence of need satisfaction, low need satisfaction does not necessarily imply the presence of need frustration.

**Table 1:** Four examples of items assessing the need for competence in the context of the workplace.

	Personal experiences	External conditions
The “bright” side of human functioning	Need satisfaction *“I feel I am very good at the things I do at work.”*	Need support *“People at work tell me I am good at what I do.”*
The “dark” side of human functioning	Need frustration *“I feel incapable of succeeding in my work tasks.”*	Need thwarting *“At work, I am prohibited from accessing information that would increase my competence for completing the tasks.”*

A second key distinction lies between scales that measure personal experiences of need fulfillment (i.e., need *satisfaction* and *frustration*) and those that assess the external conditions for need fulfillment (i.e., need *support* and *thwarting*). The latter examines the extent to which social environments either actively promote/facilitate or actively undermine/hinder individuals’ need fulfillment (Vansteenkiste and Ryan, 2013). For example, the behavior of parents, coaches, or teachers can either support or thwart psychological needs, and larger settings like schools, workplaces, or even societies may differ in their impact (Ryan et al., 2019). However, need fulfillment is not solely determined by external conditions; even in supportive environments, individuals may not always experience satisfaction, and in need-thwarting environments, frustration is not guaranteed (Deci and Ryan, 1985b). This highlights that measures of external conditions do not always predict personal experiences of need fulfillment. A potential exception could be those needs that can only be fulfilled by other people (e.g., relatedness, the need for love and belonging).

Given these ongoing debates regarding the definition, categorization, and operationalization of basic psychological needs, various types of need-focused self-assessment tools have emerged over the past decades. Especially in recent years, the interest in assessing basic/fundamental psychological need fulfillment has captivated researchers across domains, such as work, education, sports, leisure, healthcare, design, and technology. Additionally, in the past decade, several papers have provided comprehensive guidelines for designing and validating psychometric scales, alongside the development of novel statistical tools and methods.

Despite this growing interest, we have not found recent reviews dedicated to need-based scale design and development. Elson et al. (2023) recently argued that many measures in psychology are only used once or twice, an issue they referred to as “the toothbrush problem” and deemed a serious barrier to cumulative scientific progress. As one of the main reasons for this proliferation, they identified the difficulty of finding reusable measures in the large, fragmented academic literature. To address this gap, the present study aims to systematically map and review original psychometric scales^1^ developed to measure psychological need fulfillment, applicable to the general population. By reviewing scales developed over the past several decades, we aim to provide valuable information for prospective users of existing psychometric scales, as well as for developers of future scales.

This manuscript aims to accomplish two primary objectives, each corresponding to a main section of the paper. The first objective (detailed in the “Results” section) is to present a comprehensive overview of published psychometric scales from 1978 to the present. This section serves as a resource for scholars and practitioners seeking existing scales for their research. Building on this overview, we identify the key factors underlying the similarities and differences among the scales, such as their theoretical foundations, scale construction methodologies, item pool generation, and scale validation processes. These factors can guide scholars in making informed decisions about which scale to use. The second objective (reported in the “Discussion” section) is to explore these key factors in greater depth. For each factor, we provide methodological considerations to be made when developing new scales. In addition, we provide practical recommendations supplemented with references to further resources that provide more detailed guidance on specific aspects of psychometric scale development.

## 2 Methods

### 2.1 Search strategy

The systematic literature search and review was independently conducted by two researchers (TK and SH) on 21 August 2023, using Scopus, Web of Science, PubMed, and ProQuest. The following keywords were used: “need*” AND either of the words “questionnaire*” OR “assess*” OR “scal*” OR “measure*” OR “tool*” OR “techniq*” in the title, AND either of the terms “psycholog* need*” OR “basic need*” OR “fundamental need*” OR “human need*,” AND “develop*” OR “valid*” in the title, abstract, or keywords. The search string was customized to align with each search engine’s format logic, and the search scope was confined to peer-reviewed scientific papers in the English language.

### 2.2 Study selection

Papers that did not address psychological need fulfillment of a healthy general population in a social context were excluded. This entailed disregarding studies focusing on non-human needs, the needs of people in very particular situations (such as living under extreme conditions, or with a chronic or terminal disease), and studies of technology-mediated need fulfillment. Furthermore, studies that did not develop, adapt, or validate need-related self-report questionnaires were also omitted.

**Figure 1:**
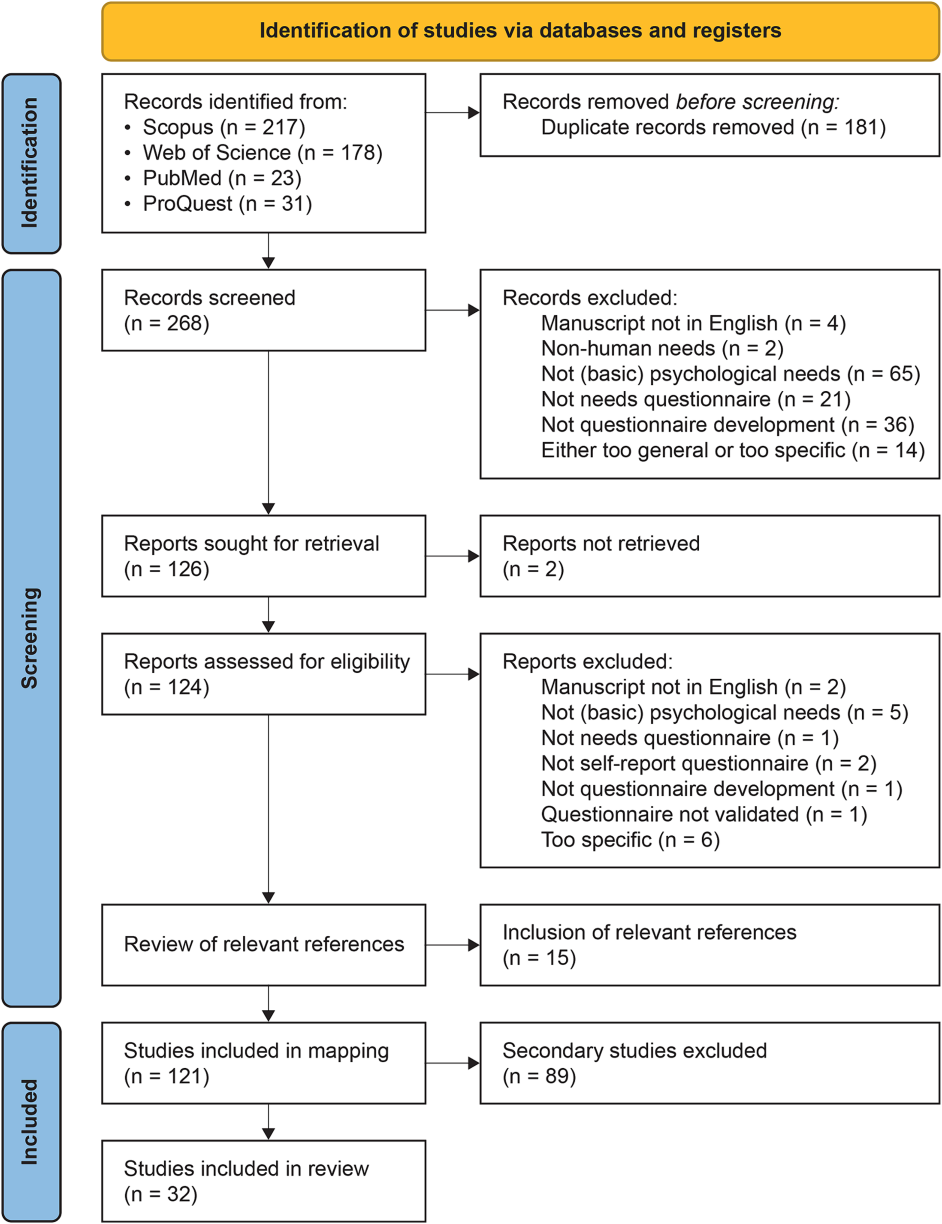
PRISMA flow diagram of literature search and study selection (template adopted from Page et al., 2021).

The resulting compilation of records was thoroughly screened to identify the studies that developed and/or validated original scales (hereinafter referred to as “primary studies”) and to distinguish them from studies that had adapted existing versions of scales (hereinafter referred to as “secondary studies”). Figure 1 offers an overview of the search and screening process. Throughout the various stages of the study selection and review process, any disagreements between the two reviewers were addressed through discussion, culminating in a consensus, with P.M.A.D. serving as a third discussion partner to resolve any remaining disagreements.

### 2.3 Data extraction and analysis

The following data were extracted from the primary studies: (1) scale name and abbreviation; (2) theoretical basis for scale development; (3) addressed states of need fulfillment; (4) application domain; (5) final construction of the scale; and study design data, including (6) item pool generation and Content analysis; (7) pilot testing; and (8) scale validation. Missing information was obtained from reliable online sources or the corresponding authors. We also recorded the citation count for all primary and secondary studies on Web of Science and Scopus on 21 August 2023.

## 3 Results

The systematic review identified 121 eligible studies, of which 32 were primary and 89 secondary. The primary studies described the development of 31 original scales, of which 23 were based on SDT, and the remaining eight were built on other (hereinafter referred to as “alternative”) theories. All identified original scales are presented in Table 2.

**Table 2:** Identified original scales (ordered alphabetically) and associated primary studies.

Scale name (Abbreviation)	Primary study
Activity-feeling states scales (AFS)	Reeve and Sickenius (1994)
Adolescent psychological need support in exercise questionnaire (APNSEQ)	Emm-Collison et al. (2016)
Adolescent students’ basic psychological needs at school scale (ASBPNSS)	Tian et al. (2014)
Balanced measure of psychological needs scale (BMPN)	Sheldon and Gunz (2009) and Sheldon and Hilpert (2012)
Basic needs satisfaction scale (BNSS)	Saeednia (2011)
Basic needs satisfaction in sport scale (BNSSS)	Ng et al. (2011)
Basic needs satisfaction at work scale (BNSW-S)	Deci et al. (2001)
Basic psychological needs in exercise scale (BPNES)	Vlachopoulos and Michailidou (2006)
Basic psychological needs scale (BPNS)	Gagné (2003)
Basic psychological need satisfaction and frustration scale (BPNSFS)	Chen et al. (2015)
Basic psychological needs scale for teachers (BPNS-T)	Alvarado et al. (2021)
Basic psychological needs at work scale (BPNWS)	Brien et al. (2012)
DRAMMA questionnaire	de Bloom et al. (2017)
Existence, relatedness, and growth needs scale (ERG)	Poulou and Norwich (2019)
General need satisfaction and frustration scale (GNSF)	Neubauer et al. (2022)
Interpersonal behaviors questionnaire (IBQ)	Rocchi et al. (2017)
Learning climate questionnaire (LCQ)	Williams (1991)
Manifest needs questionnaire (MNQ)	Steers and Braunstein (1976)
Novelty need satisfaction scale (NNSS)	Gonzalez-Cutre et al. (2016)
Need satisfaction and frustration scale (NSFS)	Longo et al. (2016)
Need-supportive teaching style scale in physical education (NSTSSPE)	Liu and Chung (2017)
Psychological need frustration scale for physical activity (PNFS-PA)	Chung et al. (2020)
Psychological need satisfaction in exercise scale (PNSE)	Wilson et al. (2006)
Psychological needs satisfaction scale in physical education (PNSSPE)	Liu and Chung (2014)
Psychological need states in sport-scale (PNSS-S)	Bhavsar et al. (2020)
Psychological need thwarting scale (PNTS)	Bartholomew et al. (2011b)
Satisfaction of psychological needs through physical activity instrument	Dunton et al. (2023)
“Sheldon scale”	Sheldon et al. (2001)
Work-related basic need satisfaction scale (W-BNS)	Van den Broeck et al. (2010)
Work motivation form - employee (WMF-E)	Kasser et al. (1992)
Work needs satisfaction scales (WNSS)	Autin et al. (2019)

### 3.1 Chronological overview

**Figure 2:**
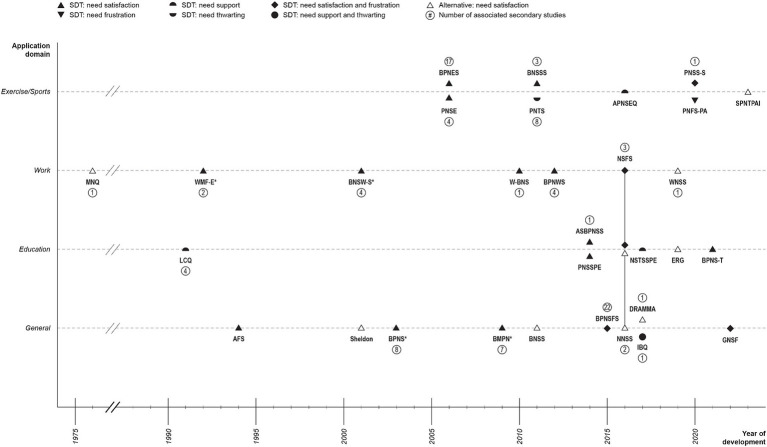
Chronological overview of the reviewed 31 original scales by domain, theoretical basis, and need fulfillment state. Slanted lines between the years 1975 and 1990 indicate a non-calibrated time gap; *related studies; AFS, Activity-feeling states scales; APNSEQ, Adolescent psychological need support in exercise questionnaire; ASBPNSS, Adolescent Students’ basic psychological needs at school scale; BMPN, Balanced measure of psychological needs scale; BNSS, Basic-needs-satisfaction scale; BNSSS, Basic needs satisfaction in sport scale; BNSW-S, Basic needs satisfaction at work scale; BPNES, Basic psychological needs in exercise scale; BPNS, Basic psychological needs scale; BPNSFS, Basic psychological need satisfaction and frustration scale; BPNS-T, Basic psychological needs scale for teachers; BPNWS, Basic psychological needs at work scale; DRAMMA, DRAMMA questionnaire; ERG, Existence, relatedness, and growth needs scale; GNSF, General need satisfaction and frustration scale; IBQ, Interpersonal behaviors questionnaire; LCQ, Learning climate questionnaire; MNQ, Manifest needs questionnaire; NNSS, Novelty need satisfaction scale; NSFS, Need satisfaction and frustration scale - educational and work contexts; NSTSSPE, Need-supportive teaching style scale in physical education; PNFS-PA, Psychological need frustration scale for physical activity; PNSE, Psychological need satisfaction in exercise scale; PNSSPE, Psychological needs satisfaction scale in physical education; PNSS-S, Psychological need states in sport-scale; PNTS, Psychological need thwarting scale; SPNTPAI, Satisfaction of psychological needs through physical activity instrument; Sheldon, “Sheldon scale”; W-BNS, Work-related basic need satisfaction scale; WMF-E, Work Motivation form-employee; WNSS, Work needs satisfaction scales.

As presented in Figure 2, the earliest identified scale dated back to 1976 and was designed to evaluate need satisfaction in the context of work (Steers and Braunstein, 1976). Fifteen years later, a scale was introduced in the context of education (Williams, 1991), followed by another in the work domain (Kasser et al., 1992). The first domain-general scale emerged in 1994 (Reeve and Sickenius, 1994), and by 2003, two more were developed (Sheldon et al., 2001; Gagné, 2003). The first scales assessing need satisfaction in exercise/sports were published in 2006 (Vlachopoulos and Michailidou, 2006; Wilson et al., 2006). It is noteworthy that approximately half of all primary studies were published after 2013, indicating an increased interest in need scales over the recent decade.

Early psychometric scales were designed to only assess the state of need *satisfaction* or *support*. In 2011, need *thwarting* was introduced by Bartholomew et al. (2011a, 2011b), followed by need *satisfaction and frustration* (Chen et al., 2015), need *support and thwarting* (Rocchi et al., 2017), and finally, need *frustration* (Chung et al., 2020). An attempt was also made to introduce the concept of need *unfulfillment;* however, the results from Bhavsar et al. (2020) failed to confirm this as a distinct dimension.

In addition to the primary studies, we identified 89 secondary studies designed to adapt 20 of the original questionnaires to target various domains or demographic groups, or to include additional dimensions (see Supplementary Table 1). In these cases, scales were translated, item descriptions were modified, and factors/items were removed or added. Furthermore, some researchers attempted to shorten the original scales, while others combined several existing scales to craft more comprehensive ones.

The number and diversity of subsequent studies partially reflects the application value of the 31 identified original scales. Although citation count is not necessarily a reliable indicator of research quality, it does serve as an indicator of scientific impact (Aksnes et al., 2019). Notably, the three most cited studies in our review, according to Web of Science and Scopus, were those describing the development of BNSW-S (with 1,045 and 1,142 citations, respectively; Deci et al., 2001), the BPNSFS (with 1,038 and 1,114 citations, respectively; Chen et al., 2015), and the “Sheldon scale” (with 819 and 1,069 citations, respectively; Sheldon et al., 2001).

### 3.2 Characteristics of 31 original scales

Characteristics of the reviewed original scales are detailed in the Supplementary Table 2 for SDT-based scales and Supplementary Table 3 for alternative theory-based scales. Key information is summarized in Table 3 below and briefly addressed in the following subsections.

**Table 3:** Key characteristics of the identified original scales (ordered alphabetically).

Scale *Application domain*	Theoretical basis *State of need fulfillment*	No. factors, items (item distribution)	Response scale type *Anchors*
AFS *General*	SDT *Satisfaction*	3, 12 (U)	5-point bipolar *Strongly disagree - Strongly agree*
APNSEQ *Exercise/Sport*	SDT *Support*	3, 9 (U)	7-point bipolar *Strongly disagree - Strongly agree*
ASBPNSS *Education*	SDT *Satisfaction*	3, 15 (U)	6-point bipolar *Strongly disagree - Strongly agree*
BMPN *General*	SDT *Satisfaction*	3, 18 (U)	5-point unipolar *No agreement - Much agreement*
BNSS *General*	Maslow’s theory *Satisfaction*	6, 68 (N)	4-point unipolar *Nothing - A lot*
BNSSS *Exercise/Sport*	SDT *Satisfaction*	5, 20 (N)	7-point unipolar *Not true at all - Very true*
BNSW-S *Work*	SDT *Satisfaction*	3, 21 (N)	5-point Likert scale^1^ *N/A*
BPNES *Exercise/Sport*	SDT *Satisfaction*	3, 12 (U)	5-point unipolar *I do not agree at all - I completely agree*
BPNS *General*	SDT *Satisfaction*	3, 21 (N)	7-point unipolar *Not true at all - Definitely true*
BPNSFS *General*	SDT *Satisfaction and frustration*	6, 24 (U)	5-point unipolar *Not true at all - Completely true*
BPNS-T *Education*	SDT *Satisfaction*	3, 7 (N)	5-point unipolar *Does not correspond at all - Absolutely corresponds*
BPNWS *Work*	SDT *Satisfaction*	3, 12 (U)	6-point bipolar *Strongly disagree - Strongly agree*
DRAMMA *General*	DRAMMA model *Satisfaction*	6, 18 (U)	5-point bipolar *Totally disagree - Totally agree*
ERG *Education*	ERG *Satisfaction*	4, 24 (U)	5-point unipolar *Definitely does not - Definitely does apply to me*
GNSF *General*	SDT *Satisfaction and frustration*	6, 18 (N)	5-point unipolar *Not at all true - Completely true*
IBQ *General*	SDT *Support and thwarting*	6, 24 (U)	7-point unipolar *Do not agree at all - Completely agree*
LCQ *Education*	SDT *Support*	1, 15 (/)	5-point unipolar *Not true - Very true*
MNQ *Work*	Murray’s theory *Satisfaction*	4, 20 (U)	7-point bipolar *Always - Never*
NNSS *General, Education (PE)*	Studies of novelty *Satisfaction*	1, 6 (/)	7-point unipolar *Not at all true - Very true*
NSFS *Work, Education*	SDT *Satisfaction and frustration*	6, 18 (U)	7-point bipolar *Strongly disagree - Strongly agree*
NSTSSPE *Education (PE)*	SDT *Support*	3, 15 (U)	7-point bipolar *Strongly disagree - Strongly agree*
PNFS-PA *Exercise/Sport*	SDT *Frustration*	3, 14 (N)	7-point bipolar *Strongly disagree - Strongly agree*
PNSE *Exercise/Sport*	SDT *Satisfaction*	3, 18 (U)	6-point bipolar *False - True*
PNSSPE *Education (PE)*	SDT *Satisfaction*	3, 10 (N)	7-point bipolar *Strongly disagree - Strongly agree*
PNSS-S *Exercise/Sport*	SDT *Satisfaction and frustration*	6, 29 (N)	7-point bipolar *Strongly disagree - Strongly agree*
PNTS *Exercise/Sport*	SDT *Thwarting*	3, 12 (U)	7-point bipolar *Strongly disagree - Strongly agree*
“Sheldon scale” *General*	Maslow’s theory and SDT *Satisfaction*	10, 30 (U)	5-point unipolar *Not at all - Very much*
SPNTPAI *Exercise/Sport*	Maslow’s theory and SDT *Satisfaction*	13, 33 (N)	9-point unipolar *Not at all - A lot*
W-BNS *Work*	SDT *Satisfaction*	3, 16 (N)	5-point bipolar *Totally disagree - Totally agree*
WMF-E *Work*	SDT *Satisfaction*	4, 15 (N)	5-point Likert *(N/A)*
WNSS *Work*	PWT *Satisfaction*	5, 20 (U)	7-point bipolar *Strongly disagree - Strongly agree*

^1^A 5-point response scale was used in the primary study; the current version of BNSW-S consists of a 7-point bipolar scale (not at all true - very true).

U, uniform item distribution; N, non-uniform item distribution; N/A, Information not available; DRAMMA, Detachment, relaxation, autonomy, mastery, meaning, and affiliation; ERG, Existence, relatedness, and growth theory; PWT, Psychology of working theory; REQ, Recovery experience questionnaire; SDT, Self-determination theory; SPNTPAI, Satisfaction of psychological needs through physical activity instrument.

#### 3.2.1 Theoretical basis and included needs

SDT was by far the most favored theoretical basis for the development of questionnaires aimed at assessing need fulfillment, accounting for 23 of the identified studies. Alternative theories that served as bases for scale development included Murray’s theory of needs, Maslow’s Hierarchy of Needs, PWT, ERG, and the DRAMMA model, each contributing to one study. Moreover, two studies were based on both SDT and Maslow’s theory, and one study was based on a combination of various sources.

The classical SDT need trio —*autonomy, relatedness,* and *competence* (ARC)— is featured in over two-thirds of the reviewed questionnaires (21 SDT-based, two alternative theory-based). All SDT-based questionnaires include the need for *autonomy,* and all but two (LCQ, NSTSSPE) include the needs for *competence* and *relatedness.* The LCQ is designed to evaluate a single need (i.e., *autonomy*), which represents the least number of needs assessed among all the questionnaires. One SDT-based scale further decomposes the need for autonomy into its facets, including *choice, internal perceived locus of causality,* and *volition,* while another adds *dependability* as an additional need. Two SDT-based scales include the need for autonomy either as the sole factor or alongside other needs, such as *involvement* and *structure.*

In contrast, the needs included in alternative scales are much more diverse. *Autonomy* is addressed in four alternative questionnaires (BMPN, DRAMMA, MNQ, WNSS), *competence* in two (BMPN, WNSS), and *relatedness* in three (BMPN, ERG, WNSS). In addition to these, over 30 other needs are included in the alternative questionnaires, NSTSSPE, and WMF-E. The satisfaction of psychological needs through physical activity instrument was developed to evaluate the most extensive range of needs – 13 in total – among all the reviewed questionnaires. These needs encompass *physical comfort, safety, social connection, esteem from others, individual esteem, learning, challenge, entertainment, novelty, creativity, mindfulness, aesthetic appreciation, and morality.* It is of note that some of the alternative needs could be construed as constituents of ARC, although they were assigned a different name. A list of all included needs within the reviewed scales is provided in Table 4.

**Table 4:** Human needs included in the reviewed scales, the number of scales including each need, and its theoretical basis.

Need	No.	Theoretical basis
Autonomy	27	SDT and ALT
Relatedness	24	SDT and ALT
Competence	23	SDT and ALT
Physical/physiological needs	3	ALT
Self-actualization/growth	3	ALT
Self-esteem	2	ALT
Affiliation	2	ALT
Esteem	2	ALT
Novelty	2	ALT
Safety	2	ALT
Security	1	ALT
Survival	1	ALT
Existence	1	ALT
Structure	1	SDT
Love and belongingness	1	ALT
Social connection	1	ALT
Popularity-influence	1	ALT
Social contribution	1	ALT
Involvement	1	SDT
Dependability	1	SDT
Morality	1	ALT
Mindfulness	1	ALT
Dominance	1	ALT
Learning	1	ALT
Need to know and to understand	1	ALT
Challenge	1	ALT
Achievement	1	ALT
Mastery	1	ALT
Creativity	1	ALT
Aesthetic appreciation	1	ALT
Entertainment	1	ALT
Pleasure-stimulation	1	ALT
Money-luxury	1	ALT
Meaning	1	ALT
Detachment	1	ALT
Relaxation	1	ALT

SDT, Self-determination theory; ALT, Alternative theories.

#### 3.2.2 Need fulfillment states across application domains

Of the 31 original questionnaires, 21 were specifically designed to assess the state of need *satisfaction*. The remaining scales had varied objectives, including the assessment of need *satisfaction and frustration* (4), need *support* (3), need *frustration* (1), need *thwarting* (1), and need *support and thwarting* (1). All the alternative questionnaires exclusively focused on assessing need satisfaction.

Six scales that targeted the work domain (BNSW-S, BPNWS, MNQ, W-BNS, WMF-E, WNSS) assessed need *satisfaction*. Four scales implemented in education assessed need *satisfaction* (ASBPNSS, BPNS-T, ERG, PNSSPE) and two assessed need *support* (LCQ, NSTSSPE). Four scales developed for exercise/sports settings assessed need *satisfaction* (BNSSS, BPNES, PNSE, satisfaction of psychological needs through physical activity instrument). Need *support*, *thwarting*, *frustration*, and *satisfaction and frustration* were each assessed by one scale (APNSEQ, PNTS, PNFS-PA, PNSS-S, respectively). Six domain-general scales focused on need *satisfaction* (AFS, BMPN, BNSS, BPNS, DRAMMA questionnaire, the “Sheldon scale”), two assessed need *satisfaction and frustration* (BPNSFS, GNSF), and one focused on need *support and thwarting* (IBQ). Two questionnaires were developed and validated across multiple domains: NSFS evaluates need *satisfaction and frustration* at work and in education, and NNSS assesses need *satisfaction* in both domain-general and physical education contexts.

#### 3.2.3 Final scale construction

Regardless of their theoretical basis, most of the original scales were established on a three-factor structure (15). Seven scales were developed with six factors, three with four factors, two with five, one with 10, and one with 13 factors. Additionally, two scales adhered to a unidimensional structure. The number of items per scale range from six to 68, with the most common number being 18 items (5), followed by 12 or 15 items (4 each), and 20 or 24 items (3 each). The distribution of items across factors is uniform in 17 cases and non-uniform in 12.

Scales used both unipolar (14) and bipolar (15) response scales. Two studies only reported using a “Likert scale” without specifying the anchors. The most frequently used number of response options was 5 or 7 (each observed in 13 scales). Furthermore, three bipolar scales consisted of 6 points, and two unipolar scales consisted of 4 and 9 points, respectively. Notably, with the exception of one frequency scale and two intensity scales, all response-scales were agreement-based.

### 3.3 Development and validation of original scales

A comprehensive analysis of the reviewed approaches to scale development and validation is provided in the Supplementary Table 4 for SDT-based scales and Supplementary Table 5 for alternative theory-based scales. A brief summary is provided below.

#### 3.3.1 Item pool generation and content analysis

##### 3.3.1.1 Approach to item generation

In the creation of item pools, 19 SDT and six alternative studies used a *deductive* approach. This involved deriving items from existing questionnaires and need-related theories. One SDT study used an *inductive* approach, which was based on information gathered from a survey of the target population. Additionally, three SDT studies and two alternative studies employed a combination of *deductive and inductive* approaches.

##### 3.3.1.2 Assessing content and face validity

*Content validity* was assessed in 15 SDT studies. In 13 of these, one to 40 experts in need psychology were consulted, while authors assessed content validity themselves in two studies. Furthermore, one study additionally used 20 non-expert members of the target population. In three studies, the *Content Validity Index (CVI)* was calculated to guide decisions regarding item retention or elimination (Bartholomew et al., 2011b; Liu and Chung, 2014; Chung et al., 2020). Two other studies relied on *Aiken’s V*, with one of them also incorporating *Cohen’s effect size indices* (Ng et al., 2011). The remaining studies employed less rigorous methods, such as expert-led in-person discussions.

Five alternative studies assessed *content validity*. For this purpose, three and six experts were engaged in two studies, one study recruited three non-expert members of the target population, while the authors assessed content validity themselves in another. The involvement of participants in content validation was not specified in one study. Notably, none of these studies used statistical approaches to assess inter-rater reliability.

*Face validity* was assessed in nine SDT studies. In six of these, two to 33 judges were drawn from the target population, and in three, one to 10 experts were engaged. Eight of these studies assessed both *content and face validity*. Additionally, three alternative studies assessed *face validity,* all of which also included the assessment of *content validity* by the same panel of judges.

#### 3.3.2 Factor validity

##### 3.3.2.1 Internal consistency reliability

Internal consistency reliability was assessed at various stages in nearly all studies, with only two exemptions (Sheldon et al., 2001; Gonzalez-Cutre et al., 2016). For this purpose, Cronbach’s Alpha (*α*) was used in 23 studies, Raykov’s Rho (*ρ*) in two, and McDonald’s *ω* total (ω_t_), Guttman split-half, Composite Reliability (CR), and ordinal CR in one study each. All alternative studies assessed internal consistency reliability using Cronbach’s α. Ten SDT and two alternative studies assessed internal consistency reliability on more than one sample.

##### 3.3.2.2 Exploratory factor analysis (EFA)

EFA was performed in 10 studies, eight of which were based on SDT and two on alternative theories. Factors were extracted by means of Principal-Axis Factoring (PAF) in seven cases, twice in combination with promax rotation, twice with direct oblimin rotation, once with oblimin rotation with Kaiser normalization, and once with equamax and an unspecified oblique rotation. Maximum Likelihood estimation (ML) with promax rotation was used in three studies; in one case, Kaiser normalization was employed. Two SDT studies used an unspecified extraction method with oblique rotation. Across all studies, *sample sizes* for EFA ranged between 185 and 646 (median 382).

##### 3.3.2.3 Principal-components analysis (PCA)

As an alternative to EFA, PCA was used in the exploratory phase of one alternative and three SDT studies, twice with varimax rotation, once with promax rotation, and once with direct oblimin rotation. *Sample sizes* ranged between 115 and 560 (median 239).

##### 3.3.2.4 Confirmatory factor analysis (CFA)

CFA was conducted in 23 (19 SDT, four alternative) studies; in 13 of these on multiple samples. A variety of software tools were employed, including IBM SPSS Amos (7), Mplus (6), EQS (5), and Lisrel (4). ML was used in nine studies (once with bootstrapped parameter estimates), Robust ML in six, and one study reported the use of MIMIC models. Six studies did not specify the method employed for CFA. The *sample sizes* for CFA ranged between 81 and 1,185 (median 371), and in one study, CFA was only performed on the same sample of respondents as EFA. In nine studies, only CFA was conducted without a prior EFA or PCA.

##### 3.3.2.5 Exploratory structural equation modeling (ESEM)

ESEM was used as an alternative to EFA and CFA in three SDT studies with robust ML; geomin rotation was opted for in two of these. All studies performed ESEM using Mplus. *Sample sizes* ranged between 301 and 605 (median 330).

##### 3.3.2.6 Model fit indices for CFA and ESEM

The most frequently used model fit indices included the Comparative Fit Index (CFI; 23 studies), Root Mean-Square Error of Approximation (RMSEA; 23 studies), Chi-square (χ^2^; 18 studies), Standardized Root Mean Square Residual (SRMR; 17 studies), and the Non-normed Fit Index (NNFI), also referred to as the Tucker Lewis Index (TLI; 15 studies).

#### 3.3.3 Other validity tests

Eleven SDT studies assessed *criterion validity* and 17 evaluated *construct validity* (for details, please see Supplementary Tables 4 and 5). Furthermore, 12 studies tested the *in*var*iance* of the measure, with nine focusing on invariance across genders, four across ages, two across samples, and single studies assessed invariance across time, cultures, languages, social agents, grades, qualifications, workplace types, income, financial and health satisfaction, sport types, and competitive levels and experiences. Six alternative studies assessed *criterion validity* (four of them on multiple samples), and six *construct validity*. *Invariance* of the measure was tested in two studies; one assessed invariance across genders and ages, and the other invariance across gender, races, social classes, and income.

## 4 Discussion

This systematic review of measures assessing basic/fundamental psychological need fulfillment identified 31 original scales, 23 based on SDT and eight on alternative theories, including Murray’s and Maslow’s theory, PWT, ERG, and the DRAMMA model. The earliest scale dates back to 1976 and the latest was published in 2023. In addition to the classical SDT needs trio, over 30 other needs are included across the reviewed scales. Two-thirds of them were designed to assess need *satisfaction*, and more recent scales included both *satisfaction and frustration*, or *support and thwarting* of needs. The number of factors ranges from one to 13, but the majority of scales were established on a three-factor structure. The approach to item generation was mostly deductive and content analysis was performed using various methods. Internal consistency reliability was assessed in all but two studies, factor analyses were performed using EFA, PCA, CFA, and ESEM, and additional validation tests were performed in some studies.

Our results resonate with the concerns raised by Elson et al. (2023) for the context of basic/fundamental need fulfillment scales, including the so-called “Jingle and Jangle fallacies” (i.e., the assumption that a measure’s name is representative of its content or what it measures) and the potential for undisclosed measurement flexibility (e.g., altering the item pool, or using unique scoring strategies without fully understanding the psychometric implications). The authors highlighted the importance of revisiting previous work to better inform the interpretation of existing findings and increase psychological measures’ future reuse potential. The present review supports this call for a closer examination of how basic/fundamental psychological needs are conceptualized, how need fulfillment states are defined, and how scales are designed and validated. We address each of these topics in the following sections.

### 4.1 Need typologies

#### 4.1.1 Conceptualization of basic/fundamental psychological needs

While there is a diversity of concepts addressed in the literature, most authors referred to them as “basic psychological needs.” A few studies use alternative terms like “psychological needs,” “basic needs,” “fundamental needs,” or simply “needs.” This variation highlights the absence of a consensus on the criteria used to classify needs as “basic/fundamental.” Such differences in terminology can create confusion, particularly when the same term is used in the name of scales based on different need typologies (e.g., the BNSSS-based on SDT-and BNSS-based on Maslow’s theory-, both claim to assess “basic needs,” but cover substantially different content).

The main differences between existing need typologies appear to lie in scholars’ position on the innateness, universality, and interrelatedness of basic psychological needs. SDT posits that basic psychological needs are innate, while other theories consider them to be learned (Murray, 1938; McClelland, 1985; Arogundade and Olunubi, 2013). If basic psychological needs are intrinsic, they should apply universally to all human beings, aligning with SDT’s perspective, which ascribes less importance to individual differences in need strength. Most contemporary research supports the idea that people across diverse cultures and regions share the same set of basic psychological needs (Miller, 1999; Martela and Sheldon, 2019).

#### 4.1.2 Attempted modifications of SDT-based need typologies

Most scales based on SDT include ARC. However, continuous efforts are observed to refine this classical trio by either introducing new candidate needs, replacing existing needs, or deconstructing needs into their constituent facets. The rationale behind introducing new candidate needs stems from the observation that the frustration of ARC alone may not adequately predict individuals’ ill-being. Consequently, there have been attempts to expand the list of basic psychological needs with, e.g., the need for *novelty* (*−variety*) (Gonzalez-Cutre et al., 2016; Bagheri and Milyavskaya, 2020), *beneficence* (Martela and Ryan, 2016), and *morality* (Prentice et al., 2019).

In some cases, researchers have adapted individual elements of ARC to suit specific research purposes. For example, Haerens et al. (2013) substituted competence with *structure*; and Liu and Chung (Liu and Chung, 2017) replaced competence and relatedness with *structure* and *involvement*. Ng et al. (2011) took a different approach by deconstructing the need for *autonomy* into its three facets to cover the unique but correlated aspects of this need. Their approach aligns with a recent conjecture that facets of a single need can represent unique aspects of the need concept, adding nuance to enhance content coverage (Vansteenkiste et al., 2020).

While there have been ongoing efforts to refine need typologies, several important challenges still need to be addressed. These include the precise definition of need facets, and the associated methodologies required for their assessment. Future research aimed at adapting existing need typologies for scale development should carefully balance eligibility criteria for basic psychological needs with the intended purpose of measurement. In turn, lessons learned from these new explorations can help refine current standards and may also generate new insights for evaluation.

### 4.2 States of need fulfillment

#### 4.2.1 States of need fulfillment in scale design

Before 2011, the development of measurement tools primarily focused on the “bright side” of need fulfillment (*satisfaction* and *support*). Consequently, almost all scales developed before 2015 adopted a one-dimensional structure. Need *satisfaction* was the first state of interest, and it remains the most extensively explored aspect to date, presumably because it has been empirically proven to predict people’s overall well-being (Bartholomew et al., 2011b). However, scholars later found that low scores on need satisfaction did not adequately capture one’s perceived state of negative experiences, stressing the importance of also measuring the “darker side” of need fulfillment (Bartholomew et al., 2011b). Sheldon and Gunz (2009) suggested the measurement of need *frustration,* as it was found to predict one’s motivation to fulfill unmet needs through particular activities.

In the past decade, there has been increasing attention to assessing the perceived disruptive influence of social contextual factors on need fulfillment, encompassing both need *support* and *thwarting* (Bartholomew et al., 2011b). In the current review, two studies evaluated need-*supportive* teaching styles or instructive behaviors as perceived by students (Williams, 1991; Liu and Chung, 2017), and one addressed both need-*supportive* and *thwarting* interpersonal behaviors (Rocchi et al., 2017). This finding is consistent with advancements in motivation research, as an increasing number of studies are delving into the multivariate environmental factors that affect one’s need fulfillment (Assor et al., 2018; Mabbe et al., 2018).

#### 4.2.2 Need satisfaction, frustration, support, and thwarting

In our review, we observed that the terms “satisfaction/frustration” and “support/thwarting” were at times used interchangeably, which could lead to confusion (e.g., Costa et al., 2015; Bhavsar et al., 2020). To ensure clarity in research findings, it is crucial to distinguish between these technical terms, especially because the states of need fulfillment they describe can co-occur (e.g., Bartholomew et al., 2011b).

According to Vansteenkiste and Ryan (2013), need thwarting occurs at the *interpersonal* level, where socializing agents (e.g., caregivers, teachers, employers) are actively antagonistic toward one’s need satisfaction. In other words, need support/thwarting involves an active and direct way of fostering/undermining one’s needs by the social environment. Conversely, need satisfaction/frustration is experienced at the *intrapersonal* level, influenced by one’s socialization experiences and genetic factors (Vansteenkiste and Ryan, 2013); an individual’s interpretation of need satisfaction/frustration may be shaped by their personality style and internalized beliefs.

Although need support typically leads to need satisfaction and wellbeing, and need thwarting typically leads to need frustration and ill-being, cross-paths can also occur (Vansteenkiste and Ryan, 2013). For example:

- *“The people in my life pressure me to do things their way”* (autonomy thwarting), but *“I do things the way I want”* (autonomy satisfaction).
- *“The people in my life give me the freedom to make my own choices”* (autonomy support), but *“I do a lot of things I do not want to do”* (autonomy frustration).
- *“The people in my life send me the message that I am incompetent”* (competence thwarting), but *“I feel confident that I can do things well”* (competence satisfaction).
- *“The people in my life tell me that I can accomplish things”* (competence support), but *“I feel incapable”* (competence frustration).

Our review also revealed concerns regarding the accuracy of some scales in capturing the intended constructs. Specifically, some items of the reviewed questionnaires appear to combine elements of both need frustration and thwarting, despite being designed to measure one or the other. For example, the items *“I feel incompetent* (frustration) *because of the things I am told* (thwarting)” or *“I doubt my ability to overcome challenges* (frustration) *because of the comments I receive* (thwarting)” (adapted from PNFS-PA) combine two constructs in a single statement. Respondents’ ratings of such double-barreled items may also be misinterpreted (e.g., disagreement with the former item could be interpreted as *“I do not feel incompetent because of the things I am told”* or as *“I feel incompetent for some other reason”*). Based on this, a comprehensive investigation into need fulfillment may necessitate a measure that encompasses both levels: the interpersonal (need support/thwarting) to guide necessary modifications in the social environment, and the intrapersonal (need satisfaction/frustration) to inform required interventions at the individuals’ personal level.

Additionally, it is important to recognize that prior to 2011, “need frustration” was considered synonymous with “low need satisfaction,” as the two-dimensional model of need fulfillment had not yet been introduced. This historical conflation has been a major source of confusion, since early references to “need frustration” do not align with how frustration is conceptualized in the two-dimensional model. Thus, caution is required when selecting instruments and interpreting research data. This caution is relevant even for scales developed after 2011. A noteworthy example is the BPNSFS scale. The original version, described in Chen (2013), was designed to measure only need satisfaction, with 24 items across three factors (one for each BPNT need), with four positively and four negatively coded items per factor. A later version was developed to assess both satisfaction and frustration. In this revision, the 12 negatively coded items were repurposed to represent need frustration, resulting in a six-factor model (see Van Der Kaap-Deeder et al., 2020). While the BPNSFS remains a widely-used and valuable tool, it is important to recognize that its original items were not developed with the more recent conceptual distinctions in mind. Consequently, some of the new items appear to better reflect low need satisfaction than true need frustration (Murphy et al., 2023). As Murphy et al. (2023, p. 129) argue, “[…] the apparent distinction between the Satisfaction and Frustration scales is likely primarily driven by item-keying direction, not by substantive content distinguishing constructs of need satisfaction and need frustration.”

When selecting or designing scales for research on psychological needs, it is crucial to critically evaluate whether the items genuinely capture the constructs of need satisfaction and frustration. The above-described examples illustrate that researchers should avoid relying solely on item-keying or earlier scale versions without ensuring that the scales accurately reflect the distinct processes of active need thwarting and low need satisfaction.

#### 4.2.3 Need dissatisfaction and unfulfillment

In 2011, Bartholomew et al. (2011a, 2011b) suggested that low scores on need satisfaction might reflect need *dissatisfaction* rather than *frustration*. Additionally, in 2020, Bhavsar et al. (2020) attempted to introduce a third state of need fulfillment, the so-called need *unfulfillment,* which they defined as “the experience of a lack of need fulfillment.”

As is the case with frustration and thwarting, the term “dissatisfaction” has sometimes been used interchangeably with the term “frustration” (Sheldon and Hilpert, 2012; Neubauer and Voss, 2018), leading to ambiguity in scale design, application, and the reporting of research results. Notably, unlike need frustration, unfulfillment results from need-indifferent interpersonal behaviors that do not actively thwart others’ needs but rather leave them overlooked or neglected (Cheon et al., 2019). Bhavsar et al. (2020) did not find empirical support for a tripartite conceptualization of need states, and Sheldon and Hilpert (Sheldon and Hilpert, 2012) suggested that the state of unfulfillment might already be included in bipolar scales designed to assess need satisfaction. To resolve this, further empirical evidence is needed to determine whether need unfulfillment genuinely impacts individuals’ wellbeing or if it merely results in disengagement, characterized by indifference or having no feelings.

### 4.3 Item number, scale polarity, item wording, and response format

#### 4.3.1 Item number

Most reviewed scales featured an equal number of items across all factors. However, justification for this approach was provided in only four studies, and it appears that the decision was based on the assumption that scales need to be “balanced” in number of items, as per Sheldon and Hilpert (2012). According to Nielsen and Kreiner (2011), a balanced number of items across subscales is only required when self-assessment tests are to be scored and interpreted by the respondents themselves, in order to make scoring and interpretation less complicated and more transparent to them. Although rules of thumb exist regarding the required number of items in a scale,^2^ Hadžibajramović et al. (2022) pointed out that general recommendations may not always be useful, as this number depends on the complexity of the measured construct.

Thurstone (1947) stressed the importance of *parsimony* in psychometric scales, advocating for the simplest possible factor structure with the minimum number of items to adequately cover the construct of interest. Typically, there are two-to four-times as many items in the initial item pool as expected in the final scale set (Streiner et al., 2014; DeVellis, 2017) to ensure that the pool reflects all aspects of the construct. To reduce the number of items to the required minimum, those that measure the same aspect of a concept as other items without contributing any additional information (i.e., “redundant items”) must be excluded. Including such items produces high (>0.40) inter-item correlations (Piedmont, 2014), which can increase internal consistency reliability, but results in very low validity of the scale (Kline, 1979). In addition, items with very low inter-item correlation (<0.2) should be excluded, as they may not represent the same content domain (Piedmont, 2014). Inter-item correlation between 0.20 and 0.40 suggests that the items are reasonably homogenous and simultaneously contain sufficiently unique variance (Piedmont, 2014) - they are reasonably “similar,” while at the same time sufficiently “dissimilar” to be retained.

One approach to generating items that thoroughly cover a construct is by identifying different facets of the concept (Oosterveld, 1996), which share a common foundation, but also present unique aspects (Vansteenkiste et al., 2020). For example, the three facets of autonomy identified by Ng et al. (2011) and included in BNSSS entail *perceived choice, internal perceived locus of causality,* and *volition;* according to Bauer and McAdams (Bauer and McAdams, 2000), competence consists of four facets: *Having an impact on self, others, and the environment*, *achieving desired goals, self-mastery,* and *independence;* and Hackmann (2019) decomposed relatedness into five facets: *Friendship, love, interpersonal dialog or sharing, connection with groups,* and *caring for or helping others.* We can also distinguish between a *giving* and a *receiving* facet of relatedness, and need fulfillment can be experienced at different levels, such as the *individual, group, societal, global,* and *universal* level (Vansteenkiste et al., 2020). Ideally, each facet of a need would be covered by one item of the scale.

Given that the complexity of the measured construct should dictate the maximum number of items per scale (Robinson, 2018), and multidimensional psychometric instruments can measure constructs of varying complexities, as demonstrated above, predetermining the number of items and adding or removing them to reach an equal number of items per subscale possibly introduces systematic error.

#### 4.3.2 Scale polarity

Need fulfillment, like many other psychological concepts, is conceived as having varying degrees of magnitude represented by a construct continuum (Jebb et al., 2021). This continuum can be either unipolar or bipolar, depending on what its endpoints represent (Jebb et al., 2021). In a unipolar scale, the lower pole represents the absence of the construct (e.g., no agreement), while in a bipolar scale, the lower pole represents the presence of an opposing construct (e.g., disagreement). It is important to distinguish between unipolar and bipolar response scales, as they can result in significantly different answer distributions (Höhne et al., 2021).

Our review revealed that early scales developed for measuring need *satisfaction* and *frustration* (e.g., W-BNS) treated these two states as the endpoints of a single continuum (Van den Broeck et al., 2010). However, with advancements in motivation research, the “bright” and “dark” sides of need fulfillment were recognized as distinctive experiences that should be examined and interpreted separately (Chen et al., 2016), because the lack of one (e.g., need *satisfaction*) does not necessarily indicate the presence of the other (e.g., need *frustration;* Vansteenkiste and Ryan, 2013).

#### 4.3.3 Item wording

This review also revealed inconsistencies in the wording of scale items designed to address different need fulfillment states. For example, ten scales assessing need *satisfaction* included some negatively-worded items that were reverse-scored. While Sheldon and Gunz (2009) interpreted these as the “absence of satisfaction,” Van den Broeck et al. (2010) suggested that they represented “need frustration.” It is worth noting that their response scales used were uni-and bipolar, respectively. We assume that negative wording in need *satisfaction* and *support* scales was used to avoid acquiescence bias, as explicitly reported by Van den Broeck et al. (2010), although earlier studies concluded that negatively-worded items reduce the validity of responses (Schriesheim and Hill, 1981) and may introduce systematic error to a scale (Jackson et al., 1993; McPherson and Mohr, 2005). According to Jebb et al. (2021), reverse-worded items are acceptable if the construct is bipolar, but contaminate measurement if it is unipolar.

It should also be stressed that it is more appropriate to use positive descriptions of need frustration rather than negative descriptions of need satisfaction in need *frustration* scales (De Vaus, 2014; Longo et al., 2016). Longo et al. (2016) pointed out the problem of negative wording in W-BNS and BNSW-S, and Murphy et al. (2023) recently deemed BPNSFS invalid for use as a measure of the dual-dimension theory precisely for this reason.

In addition to negative wording, we found that some items intended to evaluate need satisfaction/dissatisfaction actually assess need support/thwarting (e.g., “There were people telling me what I had to do” in BMPN). It is of note that such items can reduce content validity, affect factor structure of the scale (Longo et al., 2016), and lead to inaccurate interpretations of measurement results.

#### 4.3.4 Response format

The choice of response scale can importantly influence measurement results and should correspond with the continuum of the construct being measured. Approximately half of the reviewed questionnaires assessing need *satisfaction* and *support* used unipolar response scales, while the other half used bipolar scales, with four being even-numbered. It remains unclear whether the responses to these different scales can be interpreted equally. As discussed above, the lower poles of uni-and bipolar scales may not convey the same meaning. In addition, the midpoint of an odd bipolar agreement scale (“neither agree nor disagree”) and the low pole of a unipolar scale (“do not agree”) could both be interpreted as “a lack of agreement with the statement.” In the case of need *satisfaction* scales, this would imply a lack of need satisfaction. The efforts by Bhavsar et al. (2020) to include the state of need *unfulfillment* as a separate dimension may have encountered challenges precisely due to the use of a bipolar response scale for assessing all three states: *Satisfaction, frustration,* and *unfulfillment*.

Similarly, *satisfaction and frustration* of needs was assessed using both types of response scales, while *thwarting* and *frustration* were assessed using bipolar response scales, all composed of an odd number of response options. Here, again, we encounter the issue of midpoint interpretation: If the respondent neither agrees nor disagrees with their need being satisfied, this could be understood as “need unfulfillment;” but how should the midpoint on a *frustration* or *thwarting* scale be interpreted? This remains an open question that should be revisited in future research.

Labeling of response scales can also influence measurement outcomes. Traditional response scales are fully-labeled (i.e., each response point has a verbal anchor), but end-labeled scales (i.e., those with verbal anchors only at the endpoints) appear to be more common in contemporary psychometric scale development. Although end-labeling can simplify the selection of verbal anchors, it increases the difficulty of responding for participants (Darbyshire and McDonald, 2004). Fully-labeled response scales, on the other hand, tend to increase acquiescence but also reduce extreme responses, increase the clarity of reverse-coded items, and yield higher test–retest reliability (Weng, 2004; Weijters et al., 2010), making them generally preferable. It is also worth noting that Höhne et al. (2021) found comparability of answers on fully-and end-labeled unipolar scales, but not on bipolar scales, where full labeling was associated with positivity bias and end labeling with a middle response style. End-labeled unipolar and bipolar scales were found to be comparable (Höhne et al., 2021).

Another factor to consider is the direction of the response scales, meaning the position of the “negative”/“null” and “positive” poles. For example, the BNSS employs a 4-point unipolar response scale where the verbal anchor “a lot” is assigned to the numerical value “1” and “nothing” is assigned to “4.” Cases like this can confuse respondents because we usually associate larger numbers with larger quantities. Jebb et al. (2021) recommend aligning the response format with the polarity of the assessed continuum. For example, a 7-point scale is intuitively perceived as unipolar when numbered from 0 to 6 (with 0 at the lower pole representing “nothing” and 6 at the upper pole representing the largest value) and as bipolar when numbered from-3 to +3 (−3 implying a negative and + 3 a positive response at the lower and upper poles, respectively).^3^

Our review revealed that some secondary studies modified the response scale format of the primary study. For example, the original BPNSFS, which assessed need *satisfaction and frustration* in general, used a unipolar 5-point scale (1 = “not true at all” - 5 = “completely true”). However, subsequent versions for use in sport and physical education employed a unipolar 7-point response scale with the original end labels, and the “Romantic partners” version used a bipolar 7-point scale [0 = “totally disagree” - 6 = “totally agree” (Van Der Kaap-Deeder et al., 2020)]. In addition to the fact that the *lack* of agreement was represented with “1” on the unipolar scale and the *opposite* of agreement with “0” on the bipolar scale, we once again encounter the issue of comparability of responses at the lower poles across these different scales. Therefore, when using existing psychometric scales as templates for developing new ones, it is best to retain the response format of the original scale. If a different response scale seems more appropriate, the practical implications of changing it should be thoroughly examined.

Regarding the number of response options, the majority of reviewed questionnaires use 5 or 7 points, aligning with previous research indicating that response scales with 5–7 points produce most reliable results. Data quality substantially decreases when the number of response options exceeds 7 or drops below 5 (Givon and Shapira, 1984; Krosnick and Presser, 2009), as seen in the Satisfaction of Psychological Needs Through Physical Activity Instrument (9 points) and BNSS (4 points), respectively. Shorter scales within the 5–7-point range tend to induce less respondent frustration, thereby possibly increasing the response rate (Babakus and Mangold, 1992), whereas longer response scales lead to stronger scale direction effects (Yan et al., 2018). Boateng et al. (2018) recommend the use of 5 points for unipolar response scales and 7 points for bipolar scales. Among the 5-and 7-point scales included in this review (26 in total), only 17 adhered to this recommendation.

### 4.4 Reviewed domains and contexts

The specific context in which need fulfillment is experienced is one of the key points for consideration when developing a scale. The earliest scale found in this review, the MNQ, is applied to *work* settings. It is based on Murray’s system of needs, which is primarily a personality-based approach to study human motivation. In line with this, the MNQ was expressly created as a tool for use in organizational settings, serving as an instrument for selecting and assigning people appropriate roles.

The relatively late emergence of instruments tailored to educational contexts could be explained by a shift in focus from need *satisfaction/frustration* to need *support/thwarting*. In particular, the relationship between teachers’ instructional styles and students’ need fulfillment has been increasingly investigated since the 2000’s (Reeve and Jang, 2006) with the aim of enhancing student motivation and performance. Interestingly, domain-general scales were introduced after those developed for work and education settings. The domain of *exercise/sport* was the last to emerge, possibly because of the late establishment of sports psychology (i.e., 1996) and the growing interest in motivation for physical activity (Deci and Ryan, 1985a; Vallerand, 1997; Vallerand, 2007).

In addition to educational institutions, sports centers, and workplaces, it is worthwhile to examine other daily-life contexts, such as those encountered within the home environment. Hewett et al. (2017) found a significant compensatory value of the home environment for meeting needs that are under-satisfied in other areas of life. As the overall experience of need fulfillment in life often results from the interplay between segments that span various life domains, e.g., unsatisfied needs in one domain can inspire compensation in others (Petrou and Bakker, 2016; de Bloom et al., 2020), an emerging area of interest involves investigating holistic need fulfillment. This is reflected in the DRAMMA model proposed by Newman et al. (2014), which served as a basis for the reviewed DRAMMA questionnaire (de Bloom et al., 2017), as well as the newly developed Needs-based Off-job Crafting Scale (NOCS; Kujanpää et al., 2022).

### 4.5 Scale validation

The findings of this review suggest that, in general, more rigorous approaches were used to validate SDT-based scales as compared to those based on alternative theories. To date, several authors have published detailed guidelines for the development and validation of psychometric scales (e.g., Worthington and Whittaker, 2006; Roopa and Rani, 2012; Morgado et al., 2017; Mourougan and Sethuraman, 2017; Boateng et al., 2018; Kyriazos and Stalikas, 2018; Kishore et al., 2021). For in-depth guidance, we refer the reader to these resources. The following section provides a brief overview of scale-validation issues that stood out most during our review.

#### 4.5.1 Content analysis

About two-thirds of the reviewed studies assessed *content validity* of the proposed items (considering factors such as representativeness, relevance, essentiality). Around one-third of the studies assessed *face validity* (considering criteria like clarity, readability, comprehensibility, appropriateness of wording for the target population).^4^ Interestingly, quantitative approaches to content analysis were employed only in five SDT studies, four of which used an inductive approach to generate item pools. Surprisingly, quantitative methods were never used to assess face validity.

Establishing *content validity* of the proposed item pool is a vital step to support the validity of a psychometric scale (Haynes et al., 1995; Yusoff, 2019a), and insufficient *face validity* can cause the respondents to misunderstand the items, which highlights the importance of thorough content analysis. More rigorous approaches to assess *content validity* include calculating the CVI based on a sufficient number of expert opinions (Yusoff, 2019a). Similarly, *face validity* can be established using the Face Validity Index (FVI), and the panel of judges should include a sufficient number of the target respondents (Yusoff, 2019b). Alternative methods also exist, such as the use of Aiken’s V or Cohen’s effect size indices, as demonstrated by Ng et al. (2011). Although the common practice is to only present the judge panel with a description of the measured construct and a list of candidate scale items, also including the proposed response scale in the analysis could help ensure adequate interpretability and avoid issues outlined in previous sections.

#### 4.5.2 Internal consistency reliability

Among the psychometric properties assessed in the reviewed studies, internal consistency reliability was the most frequently reported. However, the interpretation of Cronbach’s coefficient *α* varied across studies, which highlights the importance of aligning interpretations with established guidelines. For example, Steers and Braunstein (1976) reported α values between 0.56 and 0.66 for three subscales of MNQ, describing these as *“acceptable […], given the type of measure.”* Similarly, Saeednia (2011) described the BNSS subscales as *“internally consistent”* with α values ranging from 0.43 to 0.65. These interpretations could benefit from further consideration of recommended thresholds. According to Ab Hamid et al. (2017), Cronbach’s α values of 0.70 or higher are generally considered indicative of good internal consistency, while values between 0.60 and 0.70 may be acceptable in exploratory research stages.

It should also be noted that a high α does not always indicate a high level of internal consistency. Very short scales, for example, often result in low α values due to their brevity, and adding more items for the same concept can increase α (Tavakol and Dennick, 2011). Williams and Woodward (1980) assumed this to be the case for MNQ, and Dreher and Mai-Dalton (1983) cautioned against using the scale in its current form. Very high Cronbach’s α values (>0.90) should, however, also be avoided, as they may suggest item redundancy (Tavakol and Dennick, 2011). For these reasons, some authors argue that Cronbach’s α cannot simply be interpreted as an index of internal consistency (Green et al., 1977; Green and Thompson, 1989; Boyle, 1991; Cortina, 1993).

#### 4.5.3 Factor validity

Of the reviewed studies, only 14 reported performing exploratory analyses to assess factorial structure. Among these, four used PCA, which is not recommended, as it may overestimate factor loadings (Costello and Osborne, 2005; Worthington and Whittaker, 2006). The most suitable EFA method for data that severely violates multivariate normality is PAF, while ML is best for normally-distributed data (Costello and Osborne, 2005; Worthington and Whittaker, 2006).

The practice of factor retention and item deletion based on Eigenvalues, though often-used, is generally considered one of the less accurate methods and is, therefore, not recommended (Costello and Osborne, 2005). For example, Sheldon et al. (2001) encountered challenges in extracting the expected 10 factors for their well-known scale using this method. They opted to proceed, explaining: *“[…] our approach does not require that all candidate needs suggested by existing theories emerge as empirically distinct […]”* This example highlights the importance of considering alternative approaches when the anticipated factor structure does not emerge clearly from the data. Despite these challenges, Sheldon’s scale has been influential, contributing to the development and validation of subsequent scales for assessing need fulfillment (e.g., ERG, BMPN). Worthington and Whittaker (2006) advise that when the desired factor structure is not adequately reproduced by EFA, researchers should either adopt the solution supported by the data or revisit item generation and earlier steps in the development process.

CFA was performed in 23 studies, in nine of these without a prior exploratory analysis, and in one study using the same sample as for EFA, which conflicts with the established guidelines. We observed that ESEM was used in three more recent studies to combine EFA and CFA; this appears to be an emerging new approach to statistical testing of psychometric scales.

Most EFAs and CFAs in the reviewed studies were performed on sufficiently large *respondent samples*. According to Worthington and Whittaker (2006), a sample size of 300 or more respondents is generally sufficient in most cases. Sample sizes of 150–200 respondents suffice when communalities are higher than 0.50, or when there are 10:1 items per factor with factor loadings at |0.4|, and 100–150 respondents may be adequate if all communalities are 0.60 or greater or when there are at least 4:1 items per factor with factor loadings larger than |0.6|. Fewer than 100 respondents or a participant-to-item ratio below 3:1 are always considered inadequate (Worthington and Whittaker, 2006). This was the case for CFA of the DRAMMA questionnaire (85 respondents), and for one of multiple CFAs of BPNS (81 respondents) and GNSF (84 respondents). Increasing the sample size can help when item communalities are low (<0.4), when there are several cross-loading items, or when there are three items per factor (Costello and Osborne, 2005).

### 4.6 Referencing issues, inconsistent scale names, and abbreviations

Throughout the review process, we encountered several challenges related to referencing practices, inconsistent scale names, and abbreviations. Although these issues may seem minor, they required substantial investigative work to trace the origins of certain scales and connect subsequent studies to their primary sources. We mention them to underscore the importance of precision and consistency in referencing and naming conventions to facilitate clarity and reduce ambiguity in the field. They include:

1. Misidentification of primary studies: In some instances, primary studies were incorrectly cited, leading to difficulties in accurate data reporting. For example, in some cases the reference for BPNWS was used when referring to W-BNS (e.g., Autin et al., 2019). Misidentifications complicate the process of understanding the evolution of scales and their proper application.
2. Inconsistent questionnaire abbreviations: Another issue was the inconsistent use of abbreviations for the same questionnaire across different studies. For instance, different abbreviations were sometimes used to refer to the same questionnaire (e.g., when referring to the questionnaire developed by Gagné (2003), subsequent studies used two different abbreviations, BPNS and BNSG-S,^5^) leading to confusion and making it difficult to track the correct references and ensure research continuity.

### 4.7 Limitations

The present study attempted to systematically review the extensive body of literature on the measurement of psychological need fulfillment across key life domains and life in general. Although we aimed to be as inclusive as possible, it is conceivable that some studies may not have been identified, given the constraints of our search criteria. A deliberate choice was made to exclude scales designed to assess need fulfillment of people facing particular circumstances. Examples are migrants, refugees, people affected by natural disasters, patients with various chronic or terminal diseases, and psychiatric disorders. These exclusions were implemented as they fell outside the scope of the present review.

## 5 Conclusion

With this systematic review, we aimed to analyze the evolutionary paths, key characteristics, and development methodologies of 31 original psychometric scales designed to assess basic/fundamental need fulfillment. Our search returned 24 eligible primary studies based on SDT and eight primary studies based on other need typologies, complemented with 89 secondary studies. Based on these, we identified the trends and issues in existing practice, and provided suggestions for future development. We focused on several core issues, including the conceptualization of basic/fundamental psychological needs, the interpretation of need fulfillment states—including the curious case of need unfulfillment—scale construction methodologies, existing and emerging domains and contexts, and the scale validation procedures. The purpose of this review was to provide a comprehensive overview of the considerations necessary when selecting or developing need assessment scales. Consequently, our discussion is broad rather than deep, setting the stage for future reviews to explore specific issues we have identified in greater detail.

One such critical issue that warrants further investigation is the distinction between need fulfillment, frustration, support, and thwarting, as well as the significant implications these distinctions hold for accurate measurement. Our review uncovered numerous factors contributing to ambiguity and confusion in this area. While these terms represent different constructs, satisfaction/frustration and supporting/thwarting have been often used interchangeably, blurring the lines between their meanings. Additionally, the historical conflation of need frustration with low need satisfaction, the overlap of cross-paths between need support, thwarting, satisfaction, and frustration, and the use of double-barreled items in several scales further complicates the interpretation of measurement results. Added to this is the confusion surrounding the concepts of need dissatisfaction and the more recent attempt to define need unfulfillment, which has introduced additional confusion in the field. If our review highlights one key point, it is the urgent need to reduce this ambiguity and confusion in order to support more precise measurements that better capture the nuanced nature of psychological need states. This will, in turn, enhance the validity of future research and contribute to a clearer understanding of need fulfillment across various contexts. Please note that we refer to the theoretical transition from the one-dimensional to the two-dimensional model of need fulfillment solely to contextualize the reviewed scales. We neither endorse nor critique this theoretical shift, nor do we take a position on which model is superior for measurement purposes. This issue remains unresolved within the field. While we acknowledge the growing body of literature advocating for the two-dimensional model, the evidence remains inconclusive, indicating that both models may offer value depending on their context of application. Further research is required to clarify these conceptual boundaries and determine which model provides the most reliable and valid assessment of psychological need fulfillment. Given the importance of this issue, it is imperative for researchers and practitioners developing or using need measurement scales to stay informed and engage with these discussions.

On the one hand, our examination of the available evidence revealed commendable efforts in developing, adapting, and applying scales to measure various states of need fulfillment within different life domains. On the other hand, it is crucial to acknowledge the existence of varying perspectives on conceptualizing psychological needs and need typologies, as well as discordant approaches in developing and validating measures, and other inconsistencies. These disparities warrant attention, as they can introduce ambiguity in the interpretation of scales and can lead to misinterpretation of research results.

By providing an overview of existing scales designed to assess need-centered experiences, this study can serve as a resource for researchers and practitioners who seek to apply such scales in their research or practice. We intentionally avoided ranking the scales, as it is not feasible to suggest that any single scale is universally superior. Instead, each scale is tailored to specific purposes and contexts, with its own set of strengths and limitations. However, our analysis of the factors that account for the differences and similarities between these scales is intended to support informed decision-making in selecting the most appropriate tool for a given research or practice context.

In addition, this overview can serve as a resource for scholars aiming to develop new or modified scales. Given the diversity of research objectives and the absence of universally accepted procedures for scale development, it is not feasible to recommend a one-size-fits-all approach. The steps required for scale development will vary depending on the intended purpose of the scale. Moreover, many factors involved in scale construction do not have a generally accepted approach. Therefore, we recommend that researchers make informed decisions for each factor, guided by our specific recommendations and suggested literature provided in this review. Furthermore, we advise that these decisions be explicitly reported during scale development process, as transparency will enhance the reproducibility and rigor of the research.

Although there is no universally recommended procedure, two of the reviewed questionnaires stood out as examples of good practice: The IBQ (Rocchi et al., 2017) and the GNSF (Neubauer et al., 2022). These scales can serve as valuable starting points for researchers new to the field. From a scale construction point of view, the IBQ uses appropriate item wording to describe need *support and thwarting,* along with an appropriate response format (i.e., a 7-point unipolar response scale). Similarly, the GNSF uses appropriate item wording to describe need *satisfaction and frustration,* as well as an appropriate response format (i.e., a 5-point unipolar response scale). Content analysis for IBQ was performed by 10 experts; for GNSF, content was analyzed multiple times, with face validity assessed by 24 members of the target population and content validity by three experts. On the negative side, neither study utilized quantitative approaches to content analysis. For factor validation, the authors of both scales opted for CFA without a prior EFA, as they based their hypothesized six-factor models on SDT. When items are expected to load onto predetermined factors with a strong theoretical basis, this approach is justified. In addition to factor validation, convergent and discriminant validity, predictive validity, and invariance across genders were also assessed for IBQ, and convergent validity, criterion validity, and invariance across ages were assessed for GNSF.

The present systematic review also offers guidance for those intending to develop new self-report measurement tools. In essence, this work ideally serves as a foundational resource that not only maps the past but also charts a course for the future of research in this vital domain of human psychology.

## Data Availability

The original contributions presented in the study are included in the article/Supplementary material, further inquiries can be directed to the corresponding author.

## Footnotes

^1^We use the terms “scale” and “questionnaire” interchangeably.

^2^According to Marsh et al. (1998), three items can yield reliable solutions; and according to Costello and Osborne (2005), five or more strongly-loading items (≥0.50) indicate a solid factor.

^3^We intentionally avoid referring to the poles as “left” and “right” due to cultural and language differences.

^4^Although face validity is often considered an element of content validity, we distinguish between the two based on the preferred type of judges: Content validity is best established by experts in the field, and face validity by the target population.

^5^For consistency, the present review adopted the name BPNS in line with most follow-up studies addressing this questionnaire.
